# Supplementary material for: Manganese Modulates Metabolic Activity and Redox Homeostasis in Translationally Blocked Lactococcus cremoris, Impacting Metabolic Persistence, Cell Culturability, and Flavor Formation
Source: Microbiol Spectr. 2022 May 31;10(3):e02708-21. doi: 10.1128/spectrum.02708-21 (PMC9241929; doi:10.1128/spectrum.02708-21)
Supplement: SUPPLEMENTAL FILE 2 — Supplemental material. Download spectrum.02708-21-s002.pdf, PDF file, 0.5 MB [file spectrum.02708-21-s002.pdf]

Supplementary material

Manganese modulates metabolic activity and redox homeostasis in translationally-blocked *Lactococcus cremoris*, impacting metabolic persistence, cell-culturability, and flavour formation.

Avis Dwi Wahyu Nugroho[1, 2, 3], Berdien van Olst[1, 3, 4], Stephanie Agnes Bachtiar[1, 2, 3], Sjef Boeren[1, 4], Michiel Kleerebezem[1, 3], Herwig Bachmann[1, 2, 5*]

Addresses:
1 TiFN, 9A, 6709 PA Wageningen, The Netherlands
2 Microbiology department, NIZO, Kernhemseweg 2, 6718 ZB Ede, The Netherlands
3 Host-Microbe Interactomics Group, Wageningen University & Research, De Elst 1, 6708 WD Wageningen, The Netherlands
4 Laboratory of Biochemistry, Stippeneng 4, 6708 WE, Wageningen University & Research, The Netherlands
5 Systems Biology Lab, Vrije Universiteit Amsterdam, De Boelelaan 1108, 1081 HZ Amsterdam, The Netherlands

Corresponding author: Bachmann, Herwig (herwig.bachmann@nizo.com)

Associated data

Supplementary Materials

Text S1: Supplementary methods, information and additional data

Text S1, PDF file, 0.5MB

Supplementary Text S1.pdf

Table S1: Complete list of proteins measured in manganese omitted compared to manganese supplemented cultures.

Table S1, XLSX file, 0.4MB

Supplementary Table S1.xlsx

Contents

Supplementary methods
1. Construction of *Lactococcus cremoris* MG1363 (pNZ5519)
2. Composition of chemically-defined medium for prolonged cultivation (CDMPC)
3. Strain-specific cultivation condition
4. Growth rate measurements
5. Proteome sample preparation and analysis
6. Fermentation end-product analysis
7. Viable cell enumeration and membrane integrity analysis

Supplementary information
1. Superoxide dismutase plays no role in stagnated acidification of translationally-blocked cells in the presence of Mn

Additional data
1. Growth characteristics of *L. cremoris* NCDO712 in the presence or absence of Mn
2. Acidification profile of translationally-blocked *L. cremoris* NCDO712 in the presence or absence of Mn

References

Supplementary methods

1. **Construction of *Lactococcus cremoris* MG1363 (pNZ5519)**

Plasmid-encoded luciferase (*luxAB*) of *Vibrio harveyi* was used as a reporter of NADH availability in *L. cremoris*. Plasmid isolation and electro-transformation were performed as described previously [1,2]. Plasmid pNZ5519 that contains the constitutive *usp45* promoter upstream of the *cre-luxAB* cassette was isolated from its cloning host *L. cremoris* NZ5500 [2] and introduced to strain MG1363 [3] .

2. **Composition of chemically-defined medium for prolonged cultivation (CDMPC) as described previously** [4]

Buffer	MW	CAS	mg/l	mM
glucose-monohydrate	198.17	14431-43-7	4950.00	24.979
Potassium phosphate monobasic	136.09	7778-77-0	2750.00	20.207
Sodium chloride	58.44	7647-14-5	2900.00	49.624
Sodium phosphate dibasic	141.96	7558-79-4	2850.00	20.076
Vitamins	MW	CAS	mg/l	uM
(±)-α-Lipoic acid or DL-6,8-Thioctic acid	206.33	1077-28-7	2.00	9.69
D-Pantothenic acid hemicalcium salt	238.27	137-08-6	0.50	2.10
Biotin	244.31	58-85-5	0.10	0.41
Nicotinic acid	123.11	59-67-6	1.00	8.12
Pyridoxal hydrochloride	203.62	65-22-5	1.00	4.91
Pyridoxine hydrochloride (Pyridoxol.HCl)	205.64	58-56-0	1.00	4.86
Thiamine hydrochloride	337.27	67-03-8	1.00	2.96
Metals	MW	CAS	mg/l	uM
Ammonium molybdate tetrahydrate	1235.86	12054-85-2	0.30	0.24
Calcium chloride dihydrate	147.02	10035-04-8	3.00	20.41
Cobalt(II) sulfate heptahydrate	281.10	10026-24-1	0.30	1.07
Copper(II) sulfate pentahydrate	249.68	7758-99-8	0.30	1.20
Iron(II) chloride tetrahydrate	198.81	13478-10-9	4.00	20.12
Magnesium chloride hexahydrate	203.30	7791-18-6	200.00	983.76
Manganese chloride tetrahydrate	197.91	13446-34-9	4.00	20.21
Zinc sulfate heptahydrate	287.56	7446-20-0	0.30	1.04
Amino acids	MW	CAS	mg/l	mM
L-Alanine	89.09	56-41-7	130	1.4592
L-Arginine	174.20	74-79-3	244	1.4007
L-Asparagine	132.12	70-47-3	80	0.6055
L-Aspartic acid	133.10	56-84-8	137	1.0293
L-Cysteine hydrochloride monohydrate	175.63	7048-04-6	61	0.3473
L-Glutamic acid	147.13	56-86-0	97	0.6593
L-Glutamine	146.14	56-85-9	96	0.6569
Glycine	75.07	56-40-6	29	0.3863
L-Histidine	155.15	71-00-1	24	0.1547

L-Isoleucine	131.17	73-32-5	82	0.6251
L-Leucine	131.17	61-90-5	117	0.8920
L-Lysine monohydrochloride	182.65	657-27-2	187	1.0238
L-Methionine	149.21	63-68-3	38	0.2547
L-Phenylalanine	165.19	63-91-2	64	0.3874
L-Proline	115.13	147-85-3	412	3.5786
L-Serine	105.09	56-45-1	172	1.6367
L-Threonine	119.12	72-19-5	68	0.5709
L-Tryptophan	204.23	73-22-3	36	0.1763
L-Tyrosine	181.19	60-18-4	50	0.27595
L-Valine	117.15	72-18-4	86	0.7341

3. Strain-specific cultivation condition

CDMPC was supplemented with lactose 30 mM for strain NCDO712 and glucose 55 m for the derivatives of strain MG1363. Chloramphenicol at final concentration of 5μg/mL and riboflavin 10 mg/L was additionally supplemented for MG1363 harbouring the luciferase encoding pNZ5519. Erythromycin at final concentration of 5μg/mL was additionally supplemented for MG1363 harbouring empty vector of pAK80 and MG1363 harbouring F1-ATPase encoding pCPC75::atpAGD. A stock of manganese chloride (2 mM) was prepared separately from the CDMPC metal supplement (in which manganese was omitted) and added into the medium to a final concentration of 20μM when indicated. Glycerol stocks of 25-generation pre-cultured strains were used for subsequent proteome and non-growing, TB-cell suspension fermentation studies.

4. Growth rate measurements

Aliquots (75μL) of *Lactococcus cremoris* NCDO712 from each serial propagation was transferred to a 384-well microtiter plate (clear). Optical density at 600 nm (OD_{600}) was measured every 30 minutes until cultures reached stationary phase. Raw data files from the microplate reader were analyzed and plotted with R (v 3.6.1). Maximum specific growth rates were calculated by determining the slope of the ln-transformed linear part of the growth curve.

5. Proteome sample preparation and analysis

Cells were harvested by centrifugation at maximum speed for 3 minutes at 4°C, dissolved in 100mM TRIS pH8 to an approximate concentration of 7.5E8 cells/ml and flash frozen. Protein isolation was performed using a modified version of the In-StageTip procedure [5]. Cell suspensions were defrosted on ice and 100µl was lysed in a water bath sonicator (Branson2510) for 5 minutes with

cooling on ice in between. The lysed culture (40µl) was loaded on StageTips containing a double Empore C18 membrane. Protein lysates were washed with 100µl 50mM ammonium bicarbonate (ABC), reduced with 20µl 20mM dithiothreitol for 45 minutes at 60°C and subsequently alkylated with 20µl 20mM acrylamide for 30 minutes at room temperature. The alkylated sample was washed with 100µl ABC and 100µl 95% ABC/5% acetonitrile. Proteins were digested overnight with 20µl 5ng/µl trypsin (Roche) at room temperature. Peptides were eluted and the membranes were washed with 70µl 1ml/L formic acid in water and with 5µl 50% acetonitrile/50% 1ml/L formic acid in water.

Mass spectra analysis was performed as described previously [6]. Raw datafiles were analyzed using MaxQuant (version 1.6.1.0) and searched against the *L. cremoris* MG1363 database (Uniprot) supplemented with NCDO712 plasmid data [7] and frequently observed contaminants. In addition to the standard settings, Trypsin/P with a maximum of two missed cleavages was set as the digestion mode, acrylamide modifications on the cysteines was set as a fixed modification, and methionine oxidation, protein N-terminal acetylation and asparagine or glutamine deamidation were set as variable modifications. A false discovery rate of 1% at protein level was allowed and the minimum required peptide length was set at 7 amino acids. At least two peptides were required to allow protein identification and quantification with at least one peptide being unique in the database.

Statistical analysis on the MaxQuant output was performed with Perseus version 1.6.2.1. Proteins were accepted when it was measured in at least 3 of the 4 replicates. For statistical analysis log10 transformed LFQ values were used and zero values were replaced by taking random values from a normal distribution with mean (measured values per biological sample -1.8) and variation (0.3 * standard deviation of the measured values per biological replicate) to enable comparative quantifications. We then performed a 2-sided two sample t-tests using the log10 normalized LFQ intensity between manganese addition and omission with a FDR threshold of 0.05 and S_0 = 0.01 [8]. Fold changes of Mn addition over Mn omission condition were calculated by dividing the log10 normalized LFQ intensity columns.

6. Fermentation end-product analysis

The concentrations of fermentation end-products in the growth media (lactic acid, formic acid, acetic acid, and ethanol) produced by strain NCDO712 were determined by high performance anion exchange chromatography (HPAEC) with UV and refractive index (RI) detection as previously

described [9]. Culture supernatant samples were collected and filtered using 0.20µm polyethersulfone (PES) membranes and stored at -20°C before analysis.

To determine the volatile compounds formed during incubation of cell suspensions in different media, a headspace solid phase micro extraction (HS-SPME) was carried out in combination with gas chromatography/mass spectrometry (Fisons, USA) as previously described [10] with a few modifications. The solid phase extraction was carried out with a grey SPME fiber (Carboxen/PDMS/Divinylbenzene; Supleco, USA) for 15 minutes at 40 °C. Subsequently, the fiber was desorbed for 3 minutes at 250°C in a splitless mode. The extracted compounds were refocused at the beginning of the column by cold trapping at -110 °C. Subsequently the trap was heated to 250°C at a rate of 50 °C/sec and the compounds were separated on a VF-Wax ms (30 m × 0.25 mm; df = 0.5 µm) capillary column (Varian, USA). The GC separation started at 40°C for 2 min, thereafter the temperature was raised with 10°C/min till 250°C and kept at 250 °C for 5 min. Mass spectral data was recorded in Full Scan mode over a range of m/z 25-250.

7. Viable cell enumeration and membrane integrity analysis

Measurements of culturable cells of NCDO712 during TB assay were performed through plating on CDMPC supplemented with 1% glucose and 0.5% UltraPure agarose (Invitrogen 16500500). Serial dilutions were prepared in PBS and 100 µL of the diluted cultures were plated on agar plates. Plates were incubated at 30°C for 24-48 hours and colonies were enumerated.

Membrane integrity of cells during prolonged incubation was analyzed using Live/Dead® BacLightTM Bacterial viability and counting kit (Invitrogen L34856) and a BD LSR Fortessa Flow Cytometry instrument (BD Biosciences), according to manufacturer instructions. A staining mixture was prepared with 1.5 µL of PI (λex/em: 535/617 nm), 1.5 µL of SYTO 9 (λex/em: 485/498 nm) stock-solutions, 5 µL microsphere standard (1E+08 beads/mL), 892 µL of running buffer (FACS Flow), and 100 µL of sample resulting in a total of 1 mL assay reaction. Fluorescence signals were measured with FITC (bandpass filter 530/30 nm) and PE-Texas Red (bandpass filter 610/20 nm) detectors. Gating was performed on the basis of fresh overnight culture (live) and cells incubated in 60% ethanol (dead). Live cells were characterized as population with high signal in FITC detector, but low signal in PE-Texas Red detector. Dead cells were characterized as the population with low signal in FITC detector, but high signal in PE-Texas Red detector. The third population of cells that was recognized displays high signal in both detectors, thereby placing these cells intermediately between

the “live” and “dead” populations. Although the viability status and physiology of these cells is not entirely clear, we classified this population as “damaged cells” based on their staining characteristics.

Additional data

1. **Growth characteristics of *L. cremoris* NCDO712 in the presence or absence of Mn**

The effect of manganese omission on the growth of our model strain NCDO712 was monitored throughout 4 subcultures (Figure S1). Growth rate, final optical density (OD600) and final pH were measured every subculture, while organic acid composition was characterized at the end of the first and the third subculture. Growth rate of the 4th subculture and organic acid composition of the 3rd subculture was shown in Figure 1 of the main manuscript.

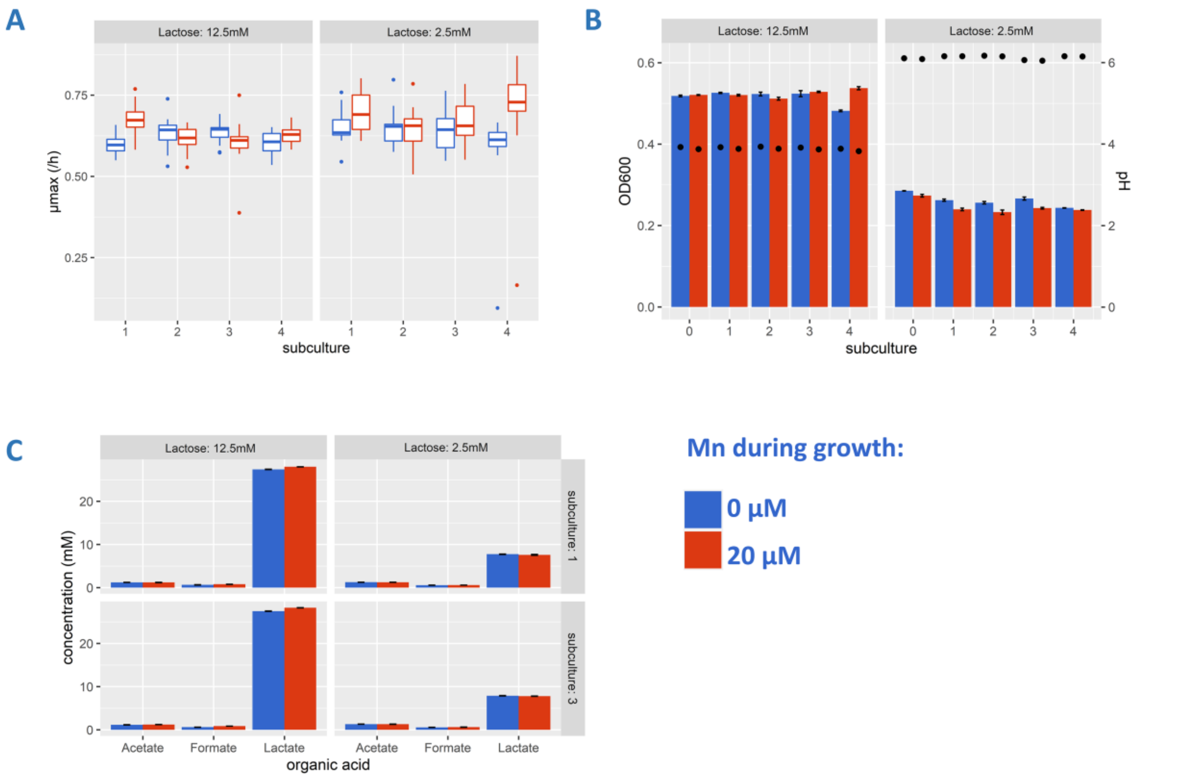

Figure S1. *Lactococcus cremoris* NCDO712 was serially propagated 4 times in defined medium supplemented with lactose at excess (12.5 mM – growth stops due to acid accumulation) or limited (2.5 mM – growth stops due to carbon depletion) concentration in the presence (red) and absence (blue) of manganese (20μM). Maximum specific growth rate (panel A) and maximum OD600 and minimum pH (panel B) were measured throughout the subcultures. Concentrations of organic acids (panel C) were measured after the 1st and the 3th subculture step. Roughly 5 generations of growth occurs in every subculture. Error bars indicate the standard deviation from 3 biological replicates, except for organic acids measurement where 2 biological replicates were analyzed.

2. **Acidification profile of translationally-blocked *L. cremoris* NCDO712 in the presence or absence of Mn**

Organic acids were measured from translationally-blocked cells of strain NCDO712. Next to lactic acid which is shown in panel A of Figure 2 of the main manuscript, formic acid and acetic acid were quantified as well. In all samples, lactic acid makes up at minimum 85% of the total acid concentrations in Cmol.

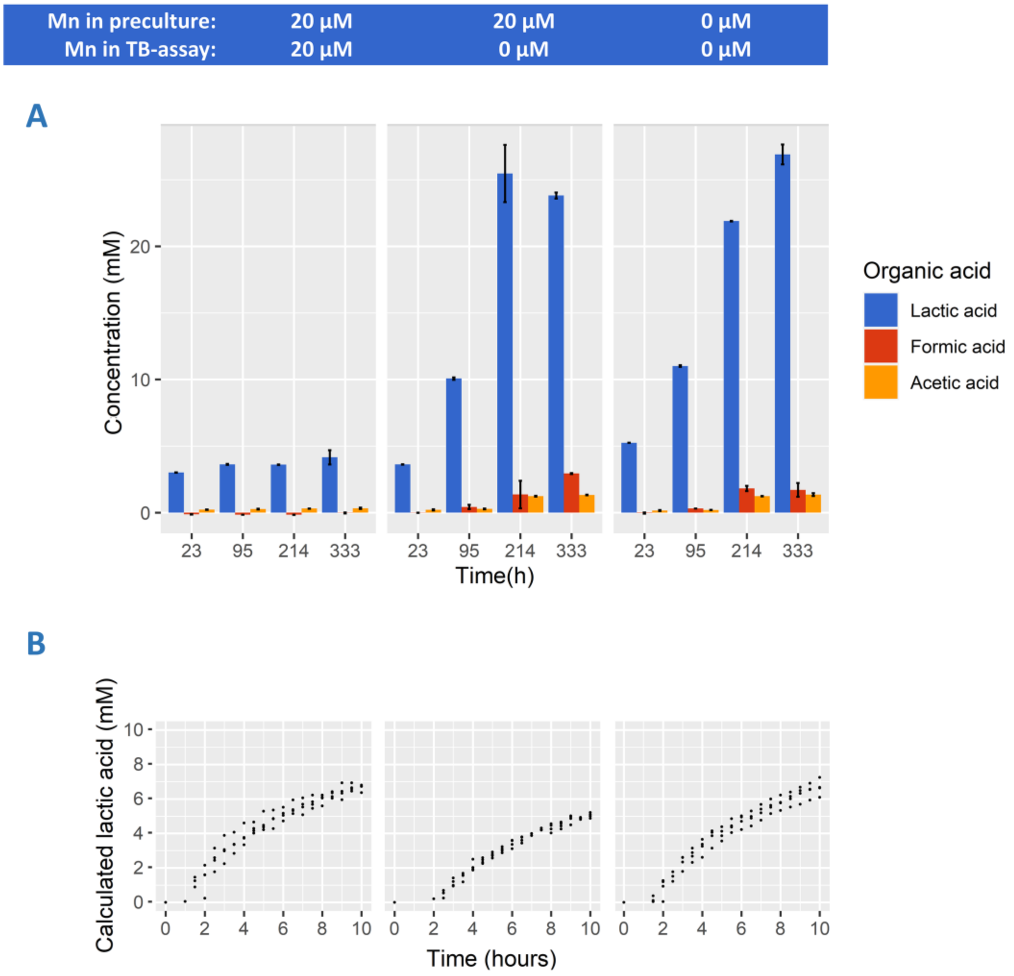

Figure S2 *Lactococcus cremoris* NCDO712 was sub-cultured 4 times in defined medium supplemented with lactose in the presence (left and middle panel) and absence (right panel) of manganese (20µM). Cells were transferred at approximately 2.5E+07 cells/mL into fresh medium containing 5µg/mL erythromycin, 30 mM lactose, and when indicated 20µM manganese (left panel). Concentration of organic acids (panel A) was measured for lactic acid (blue), formic acid (red), and acetic acid (orange) throughout incubation at 30°C. Continuous measurement of medium pH to calculate lactic acid production for the first 10 hours can be seen in panel B. Error bars indicate the standard deviation from 3 biological replicates.

Supplementary information

1. **Superoxide dismutase or oxidative stress plays no role in stagnated acidification of translationally-blocked cells in the presence of Mn**

To investigate the role of oxidative stress in relation to manganese, we employed strain NZ9000Δ*sodA* [11] that is deficient of superoxide dismutase (SOD) and thereby more sensitive to oxidative stress. As a control, we utilized the wildtype strain NZ9000 which is the host strain for the nisin controlled gene expression (NICE) system [12] and a close derivative of strain MG1363. Cells were pre-cultured in the presence of manganese and translationally-blocked assay was performed as described in the Methods section at a cell density of 2.5E+07 with glucose (55mM) as the carbon source. Acidification of SOD-deficient mutant (Figure S3) was lower than its wildtype likely due to exposure to oxygen following harvesting and assay preparation. Nonetheless, the phenotypes observed in our model strain were reproducible. Stagnated acidification was observed in the presence of manganese and cells continued to acidify in the absence of manganese. This suggest that superoxide dismutase is unlikely to be the underlying mechanism of the phenomenon described in the present study. Moreover, throughout the experiments, *L. cremoris* was incubated without aeration and with limited headspace. With different Mn supplementation, the remaining dissolved oxygen in the medium did not lead to a significant differential expression of oxidative stress response e.g. superoxide dismutase (*sodA*) in our proteomics data (Supplementary Table S1). LFQ intensity of *sodA* ranged between 7.1 and 7.4 in the absence of Mn which is a slightly lower range than between 7.3 and 7.85 when manganese is present (Supplementary Table S1).

While we do not consider oxidative stress to be a major threat in our setup, we still tried to consider the contribution of other factors that lead to oxidative stress. In *L. cremoris*, iron can be imported by *mntH* and was found to be more accumulated with higher extracellular manganese concentration, resulting in higher oxidative stress due to Fenton-type reaction [13]. Among other glycolytic enzymes, GAPDH was found to be the most sensitive to radical oxygen species (ROS) [14,15]. In this scenario, the lack of manganese would reduce iron import and the subsequent formation of ROS by Fenton-type reactions. This potentially prevents GAPDH inactivation, the eventual stagnated acidification and NADH depletion. However, our experiments with strain MG1363 showed that the absence of a plasmid-encoded *mntH* did not seem to delay the occurrence of stagnated acidification. It also has to be noted that the concentration of manganese and iron in the present study is 50- and

5-fold respectively lower than in Turner et al 2007. In combination with the absence of oxidizing agents and aeration, we consider the oxidative stress explanation to be very unlikely.

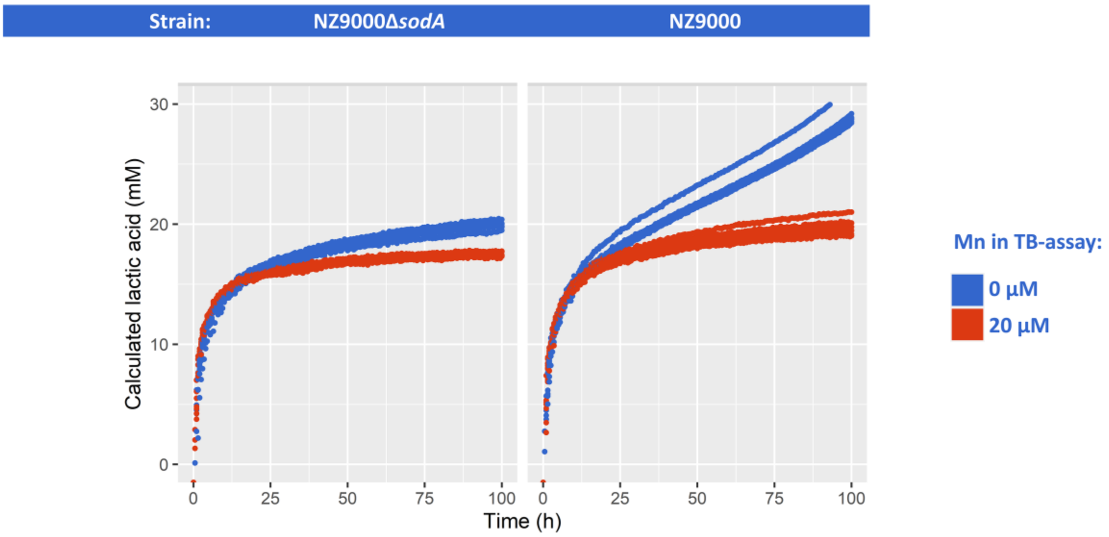

Figure S3. *Lactococcus cremoris* NZ9000 (right panel) and its derivative that contains deletion of superoxide dismutase (*sodA*) was precultured in the presence of manganese (20µM). Cells (2.5E+07 cells/mL) were transferred into fresh medium containing translational blocker erythromycin (5µg/mL) and 20µM manganese (red) or 0µM manganese (blue). Continuous measurement of medium pH to calculate lactic acid production overtime is shown. Experiment was carried out with 3 biological replicates.

References

1. Bachmann H, Santos F, Kleerebezem M, Van Hylckama Vlieg JET: **Luciferase detection during stationary phase in Lactococcus lactis**. *Appl Environ Microbiol* 2007, **73**:4704–4706.

2. Bachmann H, Kleerebezem M, Van Hylckama Vlieg JET: **High-throughput identification and validation of in situ-expressed genes of Lactococcus lactis**. *Appl Environ Microbiol* 2008, **74**:4727–4736.

3. Wegmann U, O'Connell-Motherway M, Zomer A, Buist G, Shearman C, Canchaya C, Ventura M, Goesmann A, Gasson MJ, Kuipers OP, et al.: **Complete genome sequence of the prototype lactic acid bacterium Lactococcus lactis subsp. cremoris MG1363**. *J Bacteriol* 2007, **189**:3256–3270.

4. Price CE, Branco Dos Santos F, Hesseling A, Uusitalo JJ, Bachmann H, Benavente V, Goel A, Berkhout J, Bruggeman FJ, Marrink SJ, et al.: **Adaption to glucose limitation is modulated by the pleotropic regulator CcpA, independent of selection pressure strength**. *BMC Evol Biol* 2019, **19**:15.

5. Kulak NA, Pichler G, Paron I, Nagaraj N, Mann M: **Minimal, encapsulated proteomic-sample processing applied to copy-number estimation in eukaryotic cells**. *Nat Methods* 2014, **11**:319–324.

6. Sotoca AM, Sollewijn Gelpke MD, Boeren S, Ström A, Gustafsson J-Å, Murk AJ, Rietjens IMCM, Vervoort J: **Quantitative Proteomics and Transcriptomics Addressing the Estrogen Receptor Subtype-mediated Effects in T47D Breast Cancer Cells Exposed to the Phytoestrogen Genistein**. *Mol Cell Proteomics* 2011, **10**:M110.002170.

7. Tarazanova M, Beerthuyzen M, Siezen R, Fernandez-Gutierrez MM, De Jong A, Van Der Meulen S, Kok J, Bachmann H: **Plasmid Complement of Lactococcus lactis NCDO712 Reveals a Novel Pilus Gene Cluster**. *PLoS One* 2016, **11**:e0167970.

8. Tusher VG, Tibshirani R, Chu G: **Significance analysis of microarrays applied to the ionizing radiation response**. *Proc Natl Acad Sci U S A* 2001, **98**:5116–5121.

9. Hugenholtz J, Starrenburg MJC: **Diacetyl production by different strains of Lactococcus lactis subsp. lactis var. diacetylactis and Leuconostoc spp.** *Appl Microbiol Biotechnol* 1992, **38**:17–22.

10. Gamero A, Wesselink W, de Jong C: **Comparison of the sensitivity of different aroma extraction techniques in combination with gas chromatography-mass spectrometry to detect minor aroma compounds in wine**. *J Chromatogr A* 2013, **1272**:1–7.

11. Fu RY, Bongers RS, van Swam II, Chen J, Molenaar D, Kleerebezem M, Hugenholtz J, Li Y: **Introducing glutathione biosynthetic capability into Lactococcus lactis subsp. cremoris NZ9000 improves the oxidative-stress resistance of the host**. *Metab Eng* 2006, **8**:662–671.

12. Kuipers OP, De Ruyter PGGA, Kleerebezem M, De Vos WM: **Quorum sensing-controlled gene expression in lactic acid bacteria**. *J Biotechnol* 1998, **64**:15–21.

13. Turner MS, Yu PT, Giffard PM: **Inactivation of an iron transporter in Lactococcus lactis results in resistance to tellurite and oxidative stress**. *Appl Environ Microbiol* 2007, **73**:6144–6149.

14. Cesselin B, Derré-Bobillot A, Fernandez A, Lamberet G, Lechardeur D, Yamamoto Y, Pedersen MB, Garrigues C, Gruss A, Gaudu P: **Responses of Lactic Acid Bacteria to Oxidative Stress**. In *Stress Responses of Lactic Acid Bacteria*. . Springer US; 2011:111–127.

15. Weber H, Engelmann S, Becher D, Hecker M: **Oxidative stress triggers thiol oxidation in the glyceraldehyde-3-phosphate dehydrogenase of Staphylococcus aureus**. *Mol Microbiol* 2004, **52**:133–140.
